# Analysis of the distribution of functionally relevant rare codons

**DOI:** 10.1186/1471-2164-9-207

**Published:** 2008-05-05

**Authors:** Michael Widmann, Marie Clairo, Jürgen Dippon, Jürgen Pleiss

**Affiliations:** 1 Institute of Technical Biochemistry, Allmandring 31, 70569 Stuttgart, Germany; 2 Institut für Stochastik und Anwendungen, Pfaffenwaldring 57, 70569 Stuttgart, Germany

## Abstract

**Background:**

The substitution of rare codons with more frequent codons is a commonly applied method in heterologous gene expression to increase protein yields. However, in some cases these substitutions lead to a decrease of protein solubility or activity. To predict these functionally relevant rare codons, a method was developed which is based on an analysis of multisequence alignments of homologous protein families.

**Results:**

The method successfully predicts functionally relevant codons in fatty acid binding protein and chloramphenicol acetyltransferase which had been experimentally determined. However, the analysis of 16 homologous protein families belonging to the α/β hydrolase fold showed that functionally rare codons share no common location in respect to the tertiary and secondary structure.

**Conclusion:**

A systematic analysis of multisequence alignments of homologous protein families can be used to predict rare codons with a potential impact on protein expression. Our analysis showed that most genes contain at least one putative rare codon rich region. Rare codons located near to those regions should be excluded in an approach of improving protein expression by an exchange of rare codons by more frequent codons.

## Background

The usage of codons is not random and differs between organisms and genes. Depending on the strength of an organism's translational selection, there is a bias in highly expressed genes to avoid rare codons because of the low concentration of the respective tRNA in the cell [1] which results in a decrease of translation rates [2]. As a consequence, genes with a high percentage of rare codons generally are translated at a lower rate than genes with a low percentage of rare codons [3]. Therefore, in an effort to increase the yield of recombinant proteins, rare codons have been replaced by more frequently used codons which led to increased yields of active protein [4,5].

However, gene redesign can also lead to abnormal protein folding and thus a decrease in protein solubility [6] as well as a decrease in protein activity [7,8]. It has been suggested that the differences in translational speed and the occurrence of pauses in translation is tightly linked to the folding mechanisms of the respective protein [9,10], with clustered rare codons having a greater effect on translational speed than separated rare codons [11]. Thus, optimal expression seems to be a consequence of a delicate balance between the occurrence and position of frequent and rare codons. Therefore, the effect of a replacement of rare by frequent codons to the expression level is not obvious. The goal of this work was to classify rare codons as critical and non-critical for expression of a given gene product. Non-critical rare codons could then be safely replaced by more frequent codons, while critical rare codons should not be replaced.

We suppose that critical rare codons can be predicted by comparing the codon usage of homologous proteins in a multisequence alignment. Therefore, we developed a new, cutoff independent approach to assign critical rare codons which compares the observed codon composition of one column in a multisequence alignment to all possible, alternative combinations of synonymous codons. Because the folding pathway of homologous proteins is assumed to be similar, rare codon rich regions (RCRR) which play a critical role in protein folding should be conserved in all members of a protein family. Since there is an increased probability to find rare codons in loop and linker regions [9], the location of RCRRs in respect to secondary structure elements was analyzed.

This analysis was applied to two proteins for which it was experimentally shown that an exchange of rare codons with more frequent, synonymous codons reduces activity [6,8]. The analysis of RCRRs was extended to systematically analyse a complete fold family. 16 protein families with a common α/β hydrolase fold were investigated to predict RCRRs, to localize them in respect to secondary and tertiary structure, and to identify possible RCRRs that are conserved in all members of the fold family.

## Results

### Fatty acid binding protein family

**Figure 1:**
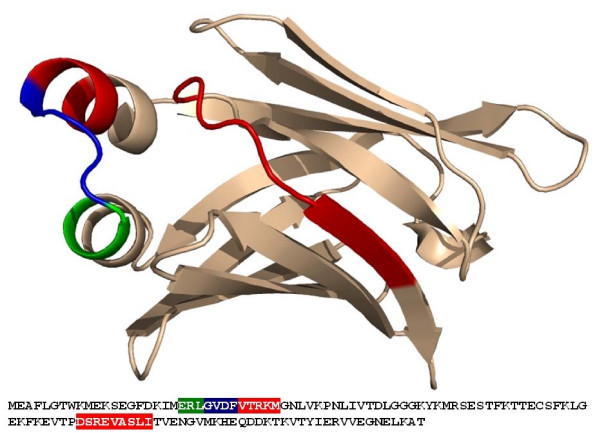
**Projection of rare codon rich regions on the sequence and the crystal structure (PDB entry 1O8V) of fatty acid binding protein**. Regions containing RCRRs are colour coded in the sequence and the three dimensional structure: a region that contains the predicted RCRRs (red), the experimentally examined region (green), a region that has been predicted and was also experimentally examined (blue).

**Figure 2:**
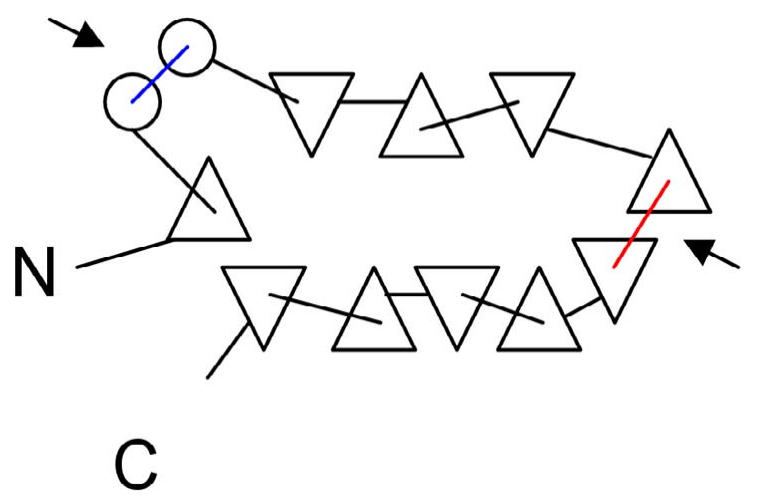
**2D projection of the fatty acid binding protein 3D structure**. View is from above towards the β-barrel. α-helices are represented as circles, β-strands as triangles. Upward and downward facing triangles represent β-strands directed upwards and downwards, respectively. Regions containing RCRRs are colour coded: a region that contains the predicted RCRRs (red) and a region that has been predicted and was also experimentally examined (blue).

A protein family of homologues to fatty acid binding protein from *E. granulosus *consisting of 10 sequences was constructed and examined for rare codon rich regions (RCRRs). Sequence identities between the sequences ranged from 82% (fatty acid binding protein from *Taenia solium *as compared to *Echinococcus granulosus*) to 37% (*Taenia solium*/*Rattus norvegicus*). Two rare codon rich regions of 9 residues each were identified in the fatty acid binding protein family with scores of 1.8 and 2.6 respectively (Fig. A1 in Additional file 1). Both RCRRs were mapped onto the 3D structure (Fig. 1) of *E. granulosus *fatty acid binding protein (PDB: 1O8V). The fatty acid binding protein belongs to the β-barrel fold family. The barrel is formed by two antiparallel β-sheets: sheet 1 (β2–β5) and sheet 2 (β6–β10 and β1) are connected by an antiparallel pair of α-helices between β1 and β2 (Fig. 2). The RCRRs are located at the connection between the two β-sheets: the first RCRR (G_24_VDFVTRKM_32_) comprises the loop connecting the two α-helices and the first turn of the second helix, the second RCRR (D_77_SREVASLI_85_) comprises the loop between strand β5 and β6 and 4 residues of the β6 strand. Previously it has been experimentally shown that the exchange of three rare codons by frequent synonymous codons in the region of the first RCRR (R_22_L_23_G_24_) leads to misfolding as concluded from a significant drop in protein solubility and induction of stress response [6].

### Chloramphenicol acetyltransferase protein family

**Figure 3:**
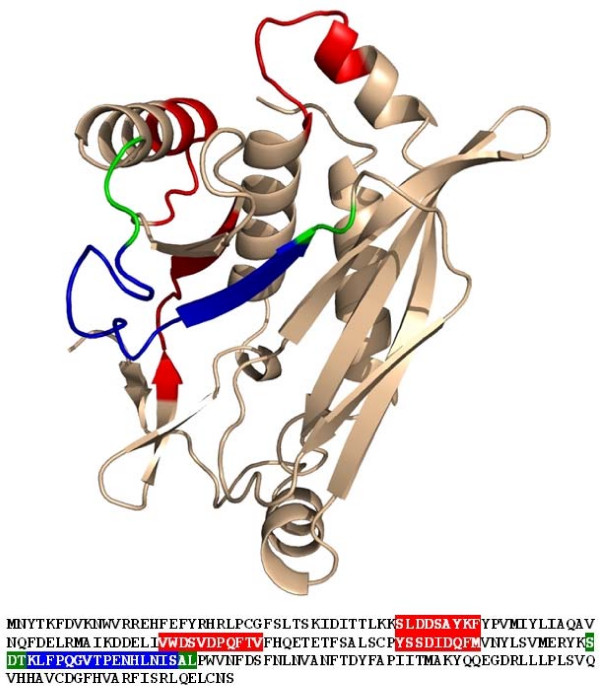
**Projection of rare codon rich regions on the sequence and the crystal structure (PDB entry 1CIA) of chloramphenicol acetyltransferase**. Regions containing RCRRs are colour coded in the sequence and the three dimensional structure: a region that contains the predicted RCRRs (red), the experimentally examined region (green), a region that has been predicted and was also experimentally examined (blue).

**Figure 4:**
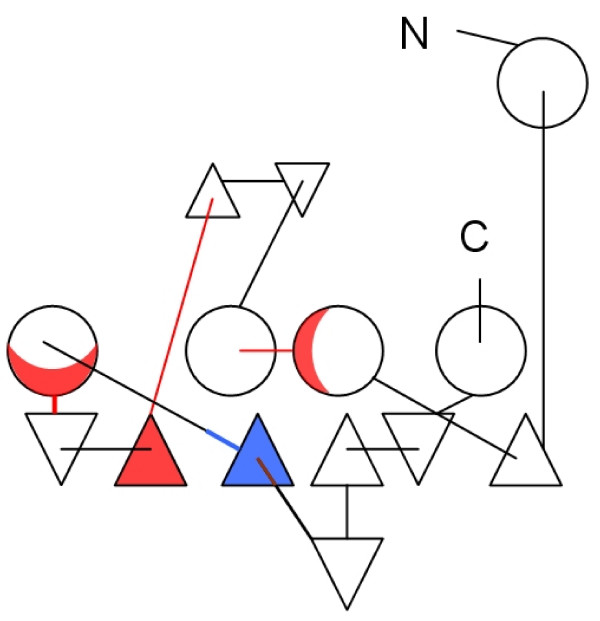
**2D projection of the chloramphenicol acetyltransferase protein 3D structure**. View is from above towards the β-barrel. α-helices are represented as circles, β-strands as triangles. Upward and downward facing triangles represent β-strands directed upwards and downwards, respectively. Regions containing RCRRs are colour coded: a region that contains the predicted RCRRs (red) and a region that has been predicted and was also experimentally examined (blue).

A protein family of homologues to chloramphenicol acetyltransferase from *M. haemolytica *consisting of 8 sequences was constructed and examined for rare codon rich regions (RCRRs). Sequence identities between the sequences ranged from 82% (chloramphenicol acetyltransferase from *Yersinia pestis biovar *as compared to *Salmonella typhimurium*) to 34% (*Enterococcus faecium*/*Salmonella typhimurium*). Four rare codon rich regions with scores of 2.8, 3.6, 2.6 and 4.8 and lengths of 9, 11, 9 and 16 respectively were identified (Fig. A2 in Additional file 2). The four RCRRs were projected on the 3D structure (Fig. 3) of the *E. coli *chloramphenicol acetyltransferase (PDB: 1CIA). The chloramphenicol acetyltransferase protein belongs to the α/β class of proteins, forming a 2-layer sandwich consisting of a β-sheet and a layer of α-helices (Fig. 4). The first RCRR is located in a loop region connecting two α-helices in the α-layer (S_42_LDDSAYKF_50_). The second RCRR is located in a long loop region leading back to the β-layer and includes the major part of a β-strand (V_79_WDSVDPQFTV_89_). The third RCRR starts in a loop connecting the β-layer and the α-layer and includes a part of a helix of the α-layer (Y_104_SSDIDQFM_112_). The fourth RCRR consists of 16 amino acids and starts in the loop connecting this helix to the next β-strand of the β-layer, including this strand (K_127_LFPQGVTPENHLNIS_142_). Previously it has been experimentally shown that the exchange of a series of rare codons by frequent synonymous codons downstream of the third RCRR and overlapping with the fourth RCRR (S_124_DTKLFPQGVTPENHLNISAL_144_) supposedly led to the elimination of a translational pause in this region and caused a drop in specific activity by 20% [8].

### α/β hydrolase families

A set of 16 homologous protein families belonging to the same α/β hydrolase fold family were systematically compared (Tab. 1). To find out whether critical rare codons are preferentially located in loop regions rather than in α-helices or β-strands, the location of RCRRs in respect to secondary structure elements was analysed. In addition, comparing the location of RCRRs in proteins with different sequence but identical fold allows to investigate whether RCRRs are conserved on the level of fold, supposing that all proteins of the same fold have a similar bottleneck in the folding pathway. Therefore, each family was examined and the RCRRs were mapped onto a crystal structure if available. 16 protein families with 7 or more proteins per family were retrieved from the Lipase Engineering Database (LED [12]) and analyzed for RCRRs. 2 protein families (abH17.01 and abH24.01) contained RCRRs but no family member with crystal structure. Therefore, the RCRRs could not be assigned to secondary structure elements. 3 families contained no RCRRs (abH09.02, abH30.01, abH31.02). 5 families only contained putative RCRRs in highly diverse regions (abH14.02, abH23.01, abH26.01, abH28.01, abH33.01). In 6 families a total of 32 RCRRs were detected and mapped to the respective crystal structure (Tab. 1). 29 RCRRs could be unambiguously assigned to one of four groups, depending on their location in secondary structure elements: (1) completely located in a loop region, (2) mainly located in a loop region (more than 50% of the RCRR in a loop region), (3) mainly located in an α-helix or a β-strand (more than 50% of the RCRR in a α-helix or a β-strand), and (4) completely located in a secondary structure element (Tab. 2). 3 RCRRs could not be assigned to a group due to missing structure information in the crystal structure. Of the 29 assigned RCRRs, 6, 8, 11, and 4 RCRRs belong to groups 1 to 4, respectively. Thus, no preference of RCRRs for loop regions was observed.

**Table 1:** Homologous protein families from the Lipase Engineering Database.

**LED ID**	**Homologous family name**	**No. of RCRRs**	**No. of sequences**	**PDB-entry**
abH01.02	*Mammalian carboxylesterases*	10	9	1K4Y
abH08.14	*Ccg1/TafII250-interacting factor B like*	2	9	1IMJ
abH09.02	*BioH protein like*	0	10	1M33
abH12.01	*Hydroxynitrile lyases*	3	10	1QJ4
abH14.02	*Gastric lipases*	0	10	1HLG
abH15.02	*Burkholderia cepacia lipase like*	6	7	4LIP
abH17.01	*Chloroflexus aurantiacus lipase like*	3	7	-
abH19.01	*Palmitoyl-protein thioesterase 1 like*	4	8	1EXW
abH23.01	*Rhizomucor mihei lipase like*	0	10	1DU4
abH24.01	*Pseudomonas lipases*	2	8	-
abH26.01	*Deacetylases*	0	7	1ODT
abH28	*Prolyl endopeptidases*	0	9	1O6F
abH30.01	*Cocaine esterases*	0	8	1L7Q
abH31.02	*Carboxymethylenebutenolidases*	0	8	1DIN
abH33.01	*Antigen 85-C*	0	10	1DQZ
abH34.02	*Serine carboxypeptidase II like*	7	9	1GXS

All families are listed with their internal unique identifier (LED ID), their family name, the number of predicted RCRRs, the number of sequences in this family, and the PDB entry used to assign predicted RCRRs to secondary structure elements.

**Table 2:** Number of predicted RCRRs in four groups of secondary structure elements.

LED ID	Group			
	1	2	3	4
abH01.02	2	2	5	-
abH08.14	1	-	-	1
abH12.01	1	1	-	1
abH15.02	-	3	1	2
abH19.01	-	1	3	-
abH34.02	2	1	2	-

Groups: (1) completely located in a loop region, (2) mainly located in a loop region (more than 50% of the RCRR in a loop region), (3) mainly located in an α-helix or a β-strand (more than 50% of the RCRR in a α-helix or a β-strand), and (4) completely located in a secondary structure element. Families are referred by their internal database identifier (LED ID, see Table 1).

**Figure 5:**
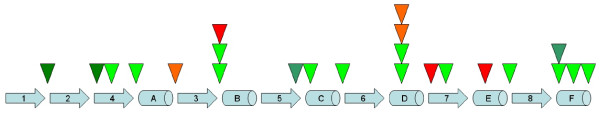
**Position and number of RCRRs, projected on a linear representation of the α/β fold**. α-helices and β-strands are depicted as cylinders and arrows, respectively, the linking loops are not shown. Predicted RCRRs are represented by coloured triangles. Each triangle represents a RCRR in one distinct homologous protein family. Triangles are coloured by the respective window score W (light green 1.8 ≤ W ≤ 2.7, dark green 2.8 ≤ W ≤ 3.7, orange 3.8 ≤ W ≤ 4.7, red 4.8 ≤ W).

To identify RCRRs that are conserved across family borders, the 32 RCRRs were mapped on the representative α/β fold and are displayed according to their respective window score (Fig. 5). Multiple RCRRs in one family in the same region were considered as only one hit. The RCRRs are distributed over 17 different positions in the representative α/β fold: 14 positions with RCRRs from only one family, 1 position with RCRRs from 2 different protein families, 1 position with RCRRs from 3 different families, and 1 position with RCRRs from 4 families. The position with RCRRs from 3 different families is located in the loop region between β-strand 3 and α-helix B. The position with RCRRs from 4 different families is located in the region of α-helix D. This region is highly variable among the protein families and often consists of more than one helix.

## Discussion

### Cutoff-independent and unbiased prediction of rare codon rich regions

In most genes an exchange of rare codons with synonymous, more frequent codons is neutral or even increases the yield of soluble protein [4,13,14]. For some genes, however, it has been observed that such an exchange surprisingly leads to an increase of incorrectly folded proteins [6,8,15]. Therefore, we based our investigation on the hypothesis that there might exist rare codons which have a regulatory function in translation and contribute to the correct folding pathway of a protein. Because the members of a homologous family and probably also of a fold family are expected to have a similar folding pathway, there should be an evolutionary bias towards the conservation of these critical rare codons. Because we only analyse synonymous codons, we restrict our analysis to the observed amino acid sequence. Thus, a possible effect to the expression level upon exchange of an amino acid is not considered by our analysis.

A rare codon is usually defined by a low usage frequency. Two types of rare codons have to be distinguished: (1) rare codons that code for an amino acid that is also encoded by more frequent codons (e.g. the arginine codon AGG) and (2) rare codons of amino acids (e.g. W,Y,H) that are encoded by only one or two rare codons. Our rare codon analysis identifies the first type of rare codons. While these rare codons are supposed to be the result of a significant evolutionary pressure towards using a rare codon instead of a frequent codon at the respective position, the second type of rare codons is strongly biased toward positions with highly conserved amino acids that are encoded exclusively by rare codons. For many organisms, codon usage tables are available [16]. However, a generally applicable distinction between rare and frequent codons is not available and the result of the analysis would depend on the choice of an arbitrary cutoff value. Therefore, we have developed a cutoff-independent approach to assign rare codons by comparing the observed codon composition of one column to all possible, alternative combinations of synonymous codons. For each column a quantitative rare codon score is derived. Instead of single columns, a sliding window of 9 columns is evaluated, because up to 27 nucleotides are involved in binding to the ribosome during translation [11] and a cumulative effect of neighbouring rare codons has been expected [17].

### Location of rare codon rich regions

It has been suggested that there is an increased tendency for rare codons in loop and linker regions [8,9,18]. For two proteins being examined for RCRRs, functionally relevant rare codons have been experimentally identified which led to a decrease of expressed active protein upon exchange by more frequent codons. Interestingly, in the gene coding for a fatty acid binding protein, the functionally relevant rare codons are located in a loop region [6], while in the second gene, the chloramphenicol acetyltransferase, the functionally relevant rare codons are located in a loop/β-strand region [8]. The observation of functionally relevant rare codons located in both loop and secondary structure regions is confirmed by our analysis of rare codon rich regions which predicts about 50% of RCRRs in loop and secondary structure regions, both in our analysis of the two experimentally examined genes and of 16 α/β hydrolase families. However, because our prediction of RCRRs is restricted to regions with a sufficient conservation of amino acids, highly diverse regions are excluded from the analysis. Therefore, functionally relevant rare codons could not be predicted if they were located in highly variable loop regions.

In the two experimentally investigated genes, RCRRs were predicted in regions linking the two halves of the β-barrel in the fatty acid binding protein and the α and β layer in the chloramphenicol acetyltransferase. Thus it is tempting to associate RCRRs with regions that link two separate folding domains. However, our systematic analysis of 16 α/β hydrolase families provides a more complex picture. Although all families are of the same fold and thus are expected to have a similar folding pathway the RCRRs are nearly equally distributed in the representative α/β hydrolase fold.

This holds true even when a more stringent cutoff is applied and RCRRs close to the minimal score requirement are eliminated. Taking all RCRRs into account, only two areas with an increased density of RCRRs are found. The region encompassing helix D with 4 RCRRs from 6 different families and the loop region connecting β-strand 3 to helix B with 3 RCRRs from 6 different families. However, the region encompassing helix D is highly variable among the α/β hydrolase families and consists of a varying number of strands and helices. The loop region connecting β-strand 3 to helix B connects the first half of the β-sheet to the second half, consisting of 4 β-strands each. Thus, there seems to be no common region in which RCRRs are located in all α/β hydrolases. In addition, 50% of all α/β hydrolase families contain no RCRRs at all. This observation can be explained by either of three possibilities: (1) There are no rare codons which are structurally conserved in all α/β hydrolases and are essential to control folding. However, RCRRs were found in individual homologous families. (2) α/β hydrolases do not have a common folding pathway. While there is evidence that proteins sharing the same fold also share a common folding pathway [19,20], this observation was based on a small set of proteins and therefore can not be generalized. Indeed, there are some studies showing that proteins sharing a common structure undergo a different folding pathway *in vitro *[21,22]. (3) The level of translational selection might differ among species. In most organisms highly expressed genes seem to contain a higher percentage of frequently used codons, while in 30% no such codon bias was found [23,24]. However, this method averages over the whole gene and therefore does not take local conservation of rare codons into account.

As it has been shown experimentally that replacing rare codons by more frequent codons in proximity to a RCRR can lead to a decrease in protein expression, the analysis of RCRRs could be helpful in predicting those critical rare codons which are probably beneficial to expression and should not be a target for codon replacement.

However, it seems that a prediction of RCRRs has to be restricted to single homologous families

## Conclusion

In most cases the substitution of rare codons with more frequent codons leads to increased protein yields in heterologous gene expression. To predict functionally relevant rare codons, multisequence alignments were analyzed to identify conserved rare codon rich regions. The prediction was validated by experimental data on silent mutations of two proteins. Therefore, we suggest that the approach of improving protein expression by an exchange of rare codons by more frequent codons should exclude rare codons located in highly conserved rare codon rich regions. A systematic analysis of 16 α/β hydrolase families predicts that most genes contain at least one putative rare codon rich region. They are however not restricted to loop regions but also occur in secondary structure elements. In addition, no preferred location of rare codon rich regions was found in respect to the common α/β hydrolase fold.

## Methods

### Protein families

Two proteins were analysed which show decreased activity upon replacement of rare by frequent codons: fatty acid binding protein from *Echinococcus granulosus *[6] and chloramphenicol acetyltransferase III from *Escherichia coli *[8].

The protein and DNA sequences of proteins homologous to fatty acid binding protein and chloramphenicol acetyltransferase III were retrieved from the GenBank by a BLAST search [25] starting with GenBank entries GenBank:Q02970 and GenBank:NP_073222, respectively. Only proteins from different organisms and with a sequence identity between 35% and 80% were selected for the subsequent multisequence alignment.

Protein and DNA sequences of 16 protein families (Tab. 1) with 7 or more proteins per family were extracted from the Lipase Engineering Database [12]. The family classification scheme of the Lipase Engineering Database was used which led to some families with overall sequence identities of only 20%. For 14 families representative structures were available in the PDB. Families with more than 10 members were reduced in size by excluding proteins from the same organism if possible, else sequences with the lowest sequence identity were removed.

A multisequence alignment of the protein sequences of each protein family was constructed using ClustalW [26] with a Gonnet Matrix [27] and a gap opening and extension penalties of 10 and 0.2, respectively. For each protein sequence, the DNA sequence was retrieved and codons were assigned to the respective amino acid in the multisequence alignment.

### Scoring method

For each column of the multisequence alignment, a codon score S was evaluated. For every amino acid, the usage frequency of its codon was taken from the Codon Usage Database [16]. These frequencies were multiplied, resulting in the column frequency α. Then all possible codon combinations were determined and their respective frequencies multiplied, resulting in codon frequencies β_i _for each combination i (i = 1,N). Each column frequency β_i _was then compared to the column frequency α, and the number n of all β_i _≤ α was determined. The score S of each column was evaluated by normalizing the number n by the number of all possible codon combinations N: S = n/N.

Small values of S correspond to a high percentage of rare codons. Thus, five groups were defined: group 1 of highly conserved rare codons with 0 ≤ S < 0.2, group 2 of conserved rare codons with 0.2 ≤ S < 0.4, group 3 with (0.4 ≤ S < 0.6), group 4 with (0.6 ≤ S < 0.8) and group 5 with (0.8 ≤ S ≤ 1). The number of columns belonging to each group was counted for each protein family and the total sum for each column group was determined (Tab. A3 in Additional file 3). From the total sums, the probability of each column group as well as the ratio between the groups was determined. To predict rare codon rich regions (RCRRs), a window of nine columns was analyzed by counting the numbers S_1 _and S_2 _of all columns belonging to group 1 and 2, respectively. The number of columns of group 1 and group 2 correspond to 2.7% and 4.5%, respectively, of all columns and have a ratio of 1.7. A window score W was evaluated by a weighted sum of S_1 _and S_2_. Because group 2 columns were 1.7 fold more frequent than group 1 columns, they were weighted with a factor of 0.6:

**W **= S_1 _+ S_2_*0.6

Thus each column of group 1 inside the window contributes a score of 1, while a column of group 2 contributes a slightly smaller score of 0.6. Areas with a window score W ≥ 1.8 are designated as a putative RCRR, beginning from the first contributing column to the last one (columns of group one or two). This score was chosen in order to avoid the detection of single columns from group 1 as a putative RCRR. Thus, a putative RCRR is predicted if at least 2 columns of group 1, 1 column of group 1 and 2 columns of group 2, or 3 columns of group 2 are found. For both cases, the probability of a random occurrence was estimated using a binominal distribution: the probability of finding 2 columns of group 1 in a window of 9 columns is 2%, and the probability of finding three or more columns of either group 1 or group 2 is 2%. Therefore, the probability of randomly finding a putative RCRR is 4%. Neighbouring RCRRs with a distance of less than 9 columns are merged. Thus, these merged RCRR will exceed the initial window length of 9 columns. Each of the putative RCRRs were evaluated for the quality of the local multisequence alignment by PLOTCON from the EMBOSS suite [28] with the EBLOSUM62 matrix. To be accepted as an RCRR the average PLOTCON score of a detected putative RCRR has to be at least 1.0. Thus, putative RCRRs that are located in highly variable regions were rejected.

## Abbreviations

RCRR: rare codon rich region.

## Authors' contributions

MW carried out the analysis and drafted the manuscript, MC contributed in developing the algorithm, JD contributed to the statistical analysis, and JP supervised the study. All authors read and approved the final manuscript.

## Supplementary Material

Additional file 1 Multisequence alignment of the fatty acid binding protein family. Click here for file

Additional file 2 Multisequence alignment of the chloramphenicol acetyltransferase protein family. Click here for file

Additional file 3 Table of the number of columns per family, sorted by groups. Click here for file
