# Supplementary material for: Analysis of the distribution of functionally relevant rare codons
Source: BMC Genomics. 2008 May 5;9:207. doi: 10.1186/1471-2164-9-207 (PMC2391168; doi:10.1186/1471-2164-9-207)
Supplement: Additional file 1 — Multisequence alignment of the fatty acid binding protein family. [file 1471-2164-9-207-S1.pdf]

```
Salmo_salar                  --MAEAFAGTWNLKDSKNFDEYMKALGVGFATRQVGGMTKPTTIIEVAGD
Cryodraco_antarcticus        --MVDVFVGTWNLKDSEKFDEYMKKLGVGFATRQVGNVTKPTTIISVEGD
Anguilla_japonica            MVIMEPFLGTWHLKTSENFDEYMKELGVGFATRKIGNTTKPTLIIAADGD
Fundulus_heteroclitus        --MVEAFVGTWNLKESENFDDYMKELGVGFATRKVGNLTKPTTIICVDGD
Tetraodon_nigroviridis       --MAEAFAGTWNLVKSEKFDEYMKELGVGLAMRKMGNLAKPTLSITIEGD
Rattus_norvegicus            --MCDAFVGTWKLVSSENFDDYMKEVGVGFATRKVAGMAKPNLIISVEGD
Danio_rerio                  --MVDKFVGTWKMTTSDNFDEYMKAIGVGFATRQVGNRTKPNLVVCVDEQ
Echinococcus_granulosus      ---MEAFLGTWKMEKSEGFDKIMERLGVDFVTRKMGNLVKPNLIVTDLGG
Taenia_solium                ---MEPFIGTWRMEKSEGFDKIMERLGVDFVTRKMGNLMKPSLIVSDLGD
Xenopus_laevis               --MVDQFVGSWKLTDSQGFDEYMQSLGVGFATRKAGAMAKPNVIISVNGD
                             xx45535454555545552445545525552555255555555555545555

Salmo_salar                  -TVTLKTQSTFKNTEISFKLGEEFDETTADDRKVKSLITIDGGKMVHVQK
Cryodraco_antarcticus        -KVTLKTQSAIKNTELSFKLDEEFDETTADDRKVKSFVTLDGGKLVHTQK
Anguilla_japonica            -KFQVKTQSLLKSTEINFKLGEEFDETTADDRKVKSVVKLEDGKLVHLQK
Fundulus_heteroclitus        -KVTVKTQSTIKNTELSFKLGEEFDETTADDRKVKSLVTIEDGKLVHVQK
Tetraodon_nigroviridis       -KVTLKNSSTFKTTEVSFKLGEEFDESTADGRNVKSVVTVEDGKLVHVQK
Rattus_norvegicus            -LVVIRSESTFKNTEISFKLGVEFDEITPDDRKVKSIITLDGGVLVHVQK
Danio_rerio                  GLICMKSQSTFKTTEIKFKLNEPFEETTADDRKTTTVMTIENGKLVQKQT
Echinococcus_granulosus      GKYKMRSESTFKTTECSFKLGEKFKEVTPDSREVASLITVENGVMKHEQD
Taenia_solium                GKYSMRSESKFKTTEFTFKLGEKFKETTPDSREVTSLITVENGVMKQEQV
Xenopus_laevis               -EILLKTESSLKTTEVTFKLGQEFDEQTADNRKTKTIITCDSGVLNQVQK
                             x55255555535555555515555345554255155531545425455445

Salmo_salar                  WDGKETTLVREVSGNALERT
Cryodraco_antarcticus        WDGKETSLVREVNGNSLTLT
Anguilla_japonica            WDSKETSLVRAVDGNKLTLT
Fundulus_heteroclitus        WDGKETTLVREVDGNKLTLT
Tetraodon_nigroviridis       WDGKETSLVREVEGNNLTLT
Rattus_norvegicus            WDGKSTTIKRRXDGDKLVVE
Danio_rerio                  WDGKESTIEREVSDGKLIAK
Echinococcus_granulosus      DKTKVTYIERVVEGNELKAT
Taenia_solium                GKGKTTYIDRVIEGNELKTT
Xenopus_laevis               WDGKETTIQREIKNGHLVVT
                             24555555552545555153
```

Figure A1

Multisequence alignment of the fatty acid binding protein family

Predicted RCRRs (red) and experimentally examined regions (green) are marked. The rare codon score S is displayed for each column.

Table A1

Organisms and GenBank identifiers of the fatty acid binding protein family

Salmo salar	GenBank: AAR91708
Cryodraco antarcticus	GenBank: AAC60357
Anguilla japonica	GenBank: BAA92355
Fundulus heteroclitus	GenBank: AAK61550
Tetraodon nigroviridis	GenBank: CAG10013
Rattus norvegicus	GenBank: NP_445817
Danio rerio	GenBank: NP_001004682
Echinococcus granulosus	GenBank: Q02970
Taenia solium	GenBank: ABB76135
Xenopus laevis	GenBank: AAH78499
