# Supplementary material for: Analysis of the distribution of functionally relevant rare codons
Source: BMC Genomics. 2008 May 5;9:207. doi: 10.1186/1471-2164-9-207 (PMC2391168; doi:10.1186/1471-2164-9-207)
Supplement: Additional file 2 — Multisequence alignment of the chloramphenicol acetyltransferase protein family. [file 1471-2164-9-207-S2.pdf]

```
Mannheimia_haemolytica        -----MNYTKFDVKNWVRREHFEFYRHRLPCGFSLTSKIDITTLKKSLDD
Aeromonas_salmonicida         -----MNFTRIDLNTWNRREHFAFYRQQIKCGFSLTTKLDITALRTALAE
Photorhabdus_luminescens      -----MNYSKVDIDLWDRKEHFLHYRNVVQCGFSLTAKIDITHLLSSLVE
Yersinia_pestis_biovar        MEKKITGYTTVDISQWHRKEHFEAFQSVAQCTYNQTVQLDITAFLKTVKK
Salmonella_typhimurium        ---------------------------------NQTVQLDITAFLKTVKK
Neisseria_meningitidis        -----MVFEKIDKNSWNRKEYFDHYFASVPCTYSMTVKVDITQIK----E
Streptococcus_suis            -----MNFNKIDLDNWKRKEIFNHYLN-QQTTFSITTEIDISVLYRNIKQ
Enterococcus_faecium          -----MTFNIINLETWDRKEYFNHYFN-QQTTYSVTKELDITLLKSMIKD
                              xxxxx314555554455551525545455434555555555345542345

Mannheimia_haemolytica        SAYKFYPVMIYLIAQAVNQFDELRMAIK-DDELIVWDSVDPQFTVFHQET
Aeromonas_salmonicida         TGYKFYPLMIYLISRAVNQFPEFRMALK-DNELIYWDQSDPVFTVFHKET
Photorhabdus_luminescens      KQYKFYPTMIYLISTVVNSYSEFRMAIK-DEELIVWDGVNPAYTIFHKET
Yersinia_pestis_biovar        NKHKFYPAFIHILARLMNAHPEFRMAMK-DGELVIWDSVHPCYTVFHEQT
Salmonella_typhimurium        NKHKFYPAFIHILARLMNAHPEFRMAMK-DGELVIWDSVHPCYTVFHEQT
Neisseria_meningitidis        KGMKLYPAMLYYIAMIVNRHSEFRTAINQDGELGIYDEMIPSYTIFHNDT
Streptococcus_suis            KRYKFYPAFVFLVTRVINSNTAFRTGYNSEGELGYWDKLDPLYTIFDSVS
Enterococcus_faecium          KGYELYPALIHAIVSVINRNKVFRTGINSEGNLGYWDKLEPLYTVFNKET
                              2255155555455555125454355145x254531354314525145353

Mannheimia_haemolytica        ETFSALSCPYSSDIDQFMVNYLSVMERYKSDTKLFPQGVTPENHLNISAL
Aeromonas_salmonicida         ETFSALSCRYFPDLSEFMAGYNAVTAEYQHDTRLFPQGNLPENHLNISSL
Photorhabdus_luminescens      ETFSAIWTEFNSDLAEFMKNYSADYETYKDDLCFFSKPELPENHFHISSV
Yersinia_pestis_biovar        ETFSSLWSEYHDDFRQFLHIYSQDIACYGENLAYFPKG-FIENMFFVSAN
Salmonella_typhimurium        ETFSSLWSEYHDDFRQFLHIYSQDVACYGENLAYFPKG-FIENMFFVSAN
Neisseria_meningitidis        ETFSSLWTECKSDFKSFLADYESDTQRYGNNHRMEGKPNAPENIFNVSMI
Streptococcus_suis            KTFSGIWTPARNDFKEFYDLYLSDVEKYNGSGKLFPKTPIPENAFSISII
Enterococcus_faecium          EKFSNIWTESNASFNSFYNSYKNDLFKYKDKNEMFPKKPIPENTVPISMI
                              54355554512354535143553545535545255251415534524154

Mannheimia_haemolytica        PWVNFDSFNLNVANFTDYFAPIITMAKYQQEGDRLLLPLSVQVHHAVCDG
Aeromonas_salmonicida         PWVSFDGFNLNITGNDDYFAPVFTMAKFQQEGDRVLLPVSVQVHHAVCDG
Photorhabdus_luminescens      PWVSFDGFNLNMASVMDYFPPIFTMGKFYQNGNQTQLPLAIQVHHATCDG
Yersinia_pestis_biovar        PWVSFTSFDLNVANMDNFFAPVFTMGKYYTQGDKVLMPLAIQVHHAVCDG
Salmonella_typhimurium        PWVSFTSFDLNVANMDNFFAPVFTMGKYYTQGDKVLMPLAIQVHHAVCDG
Neisseria_meningitidis        PWSTFDGFNLNLQKGYDYLIPIFTMGKYYKEDNKIILPLAIQVHHAVCDG
Streptococcus_suis            PWTSFTGFNLNINNNSNYLLPIITAGKFINKGNSIYLPLSLQVHHSVCDG
Enterococcus_faecium          PWIDFSSFNLNIGNNSRFLLPIITIGKFYSKDDKIYLPFSLQVHHAVCDG
                              55555535354555545554153535554545543545555445555355

Mannheimia_haemolytica        FHVARFISRLQELCNSKLK---
Aeromonas_salmonicida         FHAARFINTLQLMCDNILK---
Photorhabdus_luminescens      FHVGRVINNLQELCNDFI----
Yersinia_pestis_biovar        FHVGRMLNELQQYCDEWQGGA-
Salmonella_typhimurium        LHVGRMLNELQQYCDEWQ----
Neisseria_meningitidis        FHICRFVNELQELINS------
Streptococcus_suis            YHAGLFMNSIQELADRPNDWLF
Enterococcus_faecium          YHVSLFMNEFQNIIDNVNEWI-
                              1545554445453242xxxxxx
```

Figure A2

Multisequence alignment of the chloramphenicol acetyltransferase protein family

Predicted RCRRs (red) and experimentally examined regions (green) are marked. The rare codon score S is displayed for each column.

Table A2

Organisms and GenBank identifiers of the chloramphenicol acetyltransferase protein family

Mannheimia haemolytica	GenBank: NP_073222
Aeromonas salmonicida	GenBank: CAD57199
Photorhabdus luminescens	GenBank: NP_929686
Yersinia pestis biovar	GenBank: ZP_01174255
Salmonella typhimurium	GenBank: ABA56511
Neisseria meningitidis	GenBank: AAC14400
Streptococcus suis	GenBank: BAC11901
Enterococcus faecium	GenBank: NP_863168
