# Supplementary material for: Analysis of the distribution of functionally relevant rare codons
Source: BMC Genomics. 2008 May 5;9:207. doi: 10.1186/1471-2164-9-207 (PMC2391168; doi:10.1186/1471-2164-9-207)
Supplement: Additional file 3 — Table of the number of columns per family, sorted by groups. [file 1471-2164-9-207-S3.pdf]

Table A3

Number of columns per family, sorted by groups S1 to S5, depending on their score S. Groups: S1 with ($0 \leq S < 0.2$), S2 with ($0.2 \leq S < 0.4$), S3 with ($0.4 \leq S < 0.6$), S4 with ($0.6 \leq S < 0.8$) and S5 with ($0.8 \leq S \leq 1$)

LED ID	1	2	3	4	5
abH01.02	29	47	60	93	336
abH08.14	4	8	20	35	143
abH09.02	0	13	16	51	173
abH12.01	6	20	26	51	157
abH14.02	8	17	38	71	273
abH15.02	18	22	51	74	149
abH17.01	11	14	22	44	140
abH19.01	17	29	52	55	146
abH23.01	4	8	24	48	219
abH24.01	15	20	33	41	502
abH26.01	6	10	29	53	227
abH28	6	8	19	68	602
abH30	1	6	20	43	522
abH31.02	0	4	5	17	208
abH33.01	4	14	16	47	272
abH34.02	26	28	59	105	268
Chloramphenicol acetyltransferase protein family	15	13	24	43	115
Fatty acid binding protein family	4	9	5	17	82
Total	174	290	519	956	4534
